# Hematological parameters in patients with recurrent Aphthous Stomatitis: a systematic review and meta-analysis

**DOI:** 10.1186/s12903-024-04072-5

**Published:** 2024-03-16

**Authors:** Tahoora Mousavi, Hossein Jalali, Mahmood Moosazadeh

**Affiliations:** 1 Molecular and Cell Biology Research Center, Hemoglobinopathy Institute, Faculty of Medicine, Mazandaran University of Medical Sciences, Sari, Iran; 2 Thalassemia Research Center, Mazandaran University of Medical Sciences, Sari, Iran; 3 Epidemiology, Gastrointestinal Cancer Research Center, Non-Communicable Diseases Institute, Mazandaran University of Medical Sciences, Sari, Iran

**Keywords:** Hematological parameters, Recurrent Aphthous Stomatitis, Meta-analysis

## Abstract

**Objectives:**

Recurrent Aphthous Stomatitis (RAS) known as recurrent aphthous ulcer is a common and painful ulcerations in oral cavity. It has been suggested that hematological parameters seems to be considered as an etiologic factor. So, this meta-analysis and systematic review was aimed to examine the relationship between RAS and hematological parameters.

**Methods:**

Relevant studies were found using online international databases including Scopus, Science direct, Web of science (ISI), PubMed, and Google Scholar search engine between 2000 and October 2023. The quality of all papers was determined by NOS checklist. Heterogeneity between the results of primary studies was evaluated with I-square index and publication bias was performed by Egger’s test and funnel plots. Also, sensitivity analysis was done to check the effect of each of the primary studies on the overall estimate. Also, the statistical analyses were done using Stata software Ver. 11.

**Results:**

By combining the results of primary studies, the standardized mean difference (SMD) of vitamin B12, ferritin, folic acid, hemoglobin, iron and zinc indices with a 95% confidence interval (CI) between the case (patients with RAS) and control (Healthy) groups were estimated -0.52(-0.89, -0.14), -0.20(-0.51, 0.11), -0.42(-0.95, 0.11), -0.58(-0.90, -0.27), 0.01(-0.12, 0.15), -0.33(-0.81, 0.14) respectively. The patients with vitamin B12, ferritin, folic acid, and iron deficiencies and reduced hemoglobin (Hb) level reported 2.93(2.28, 3.78), 2.50(1.48, 4.22), 1.51(0.53, 4.29), 1.46(0.70, 3.03), and 2.14(1.38, 3.32), times more susceptible to develop RAS than healthy individuals.

**Conclusion:**

The results of the meta-analysis indicated that the SMD of vitamin B12 serum and Hb levels in the case group was 52%. Our result have also showed that the odds ratio of vitamin B12, ferritin deficiencies, and decreased Hb level in case group was 2.93, 2.50, and 2.14 times more than healthy group.

## Introduction

Recurrent Aphthous Stomatitis (RAS) is one of the most frequent ulcerative lesions with the worldwide general prevalence rate of 20%. The incidence rate of the disease is varied between 0.5% to 75% all around the world [1, 2]. RAS is more common in women and the ulcers usually begin during the second decade of life [3]. Recurrent and painful ulcerations of the oral non-keratinized mucosa are the most common clinical manifestation of this inflammatory disease [2]. All forms of RAS have a serious effect on quality of life and daily activities. The painful ulcers can interfere with eating for several days [4]. Based on the type of ulcers, the RAS is categorized into three clinical types; minor, major, and herpetiform ulcers [2]. Minor form of RAS that is also known as Miculiz’s aphthae or mild aphthous ulcers is the most common type of the diseases. The size of the created ulcers varies from 8 to 10 mm and it is frequently observed in the non-keratinized mucosal surfaces like labial mucosa, buccal mucosa, and floor of the mouth. The ulcers usually heal within 10–14 days without scarring and the diameter of ulcers is greater than 1 cm and they can persist for weeks. The major ulcers which is also called Sutton’s disease affects about 10–15% of the patients [2, 5]. Herpetiform RAS creates the greatest number of oral lesions with the most frequent recurrent oral ulcers, recurrent aphthous ulcers, or simple or complex apotheosis. The oral ulcerative lesions vary from 1 to 3 mm in diameter and in some cases they may coalesce into larger irregular ulcerations [1, 6].

The exact etiology of RAS is not well understood. However, many predisposing factors such as smoking, immunological factors, stress, hematological disorders, hormonal imbalance, infections, vitamin deficiencies, genetic factors, Helicobacter pylori and viral infections play a role in the pathogenesis of RAS [7]. It is suggested that hematological parameters particularly serum iron, folate or vitamin B12 deficiencies seems to be considered as an etiologic factor [4, 8]. Considering variable results published form different studies that have assessed the relationship between RAS and hematological parameters, combining the results of these primary studies using systematic review and meta- analysis methods can solve such controversies. So, this meta-analysis and systematic review was aimed to examine the relationship between RAS and hematological parameters.

## Material and methods

This study was designed and conducted based on Preferred Reporting Items for Systematic Reviews and Meta-Analyses (PRISMA) guidelines, but its protocol was not registered. The primary outcome was the relationship between RAS and hematological parameters.

### Search strategy

In the present study, the published articles were collected between October 2000 and 2023 through four databases such as Science direct, Scopus, Pubmed, Web of Science, and Google Scholar search engine.

Our search terms were included “Aphthous Stomatitis”, “Hematological Parameters”, “Zinc”, “Iron”, “B12”, “Folic Acid”, “Ferritin”, “Hemoglobin” with combination “OR”, “AND” and “NOT” Boolean Operators in the Title/Abstract/Keywords field (Table 1). References list of all related studies was also reviewed for any other related publications. The search was restricted to original Articles/Abstracts published in the English language that reported hematological parameters in patients with Recurrent Aphthous stomatitis. Screening, data extraction, study selection, data extraction, and quality assessment were done by two authors (HJ and TM) and any disagreements with article selection were resolved through discussion, and a third author (MM) was available to resolve the disagreement. In addition, the article references were screened to find additional related studies and increase search sensitivity. Finally, all collected references are entered into reference management software (EndNote).

**Table 1:** Literature search strategy

(Aphthous Stomatitis[MeSH Terms] ) OR (“Haematological Parameters” [Text Word]) OR (“Zinc” [Text Word]) OR(“B12”[Text Word]) OR (“Iron” [Text Word]) OR (“Folic Acid” [Text Word]) OR (“Ferritin” [Text Word]) OR (“HB” [Text Word]) OR (“Hemoglobin” [Text Word]) ; AND (Aphthous Stomatitis[Text Word]) AND (Haematological Parameters[MeSH Terms]) OR (Aphthous Stomatitis[Text Word]) AND Zinc [MeSH Terms])]) OR (Aphthous Stomatitis[Text Word]) AND ( Iron [MeSH Terms]) OR (Aphthous Stomatitis[Text Word]) AND ( B12[MeSH Terms]) OR (Aphthous Stomatitis[Text Word]) AND ( Folic Acid [MeSH Terms]) OR (Aphthous Stomatitis[Text Word]) AND ( Ferritin [MeSH Terms]) OR (Aphthous Stomatitis[Text Word]) AND ( HB [MeSH Terms]) OR (Aphthous Stomatitis[Text Word]) AND ( Hemoglobin [MeSH Terms]) AND (Filters: Publication date up to Sep 2023) AND ( Filters: English Language)	

### Inclusion criteria

The PI(E)CO process (Population, Exposure, Comparison, and Outcomes) was adopted to define inclusion and exclusion criteria for study selection. “P” signifies patients with RAS (case group); “E” expose with hematologic parameters; “C” indicates healthy group (control group). “O” means the evaluation of hematological parameters in the case and control groups.

All case–control, cross sectional, and cohort studies were entered into this meta-analysis for evaluating the hematologic status.

### Exclusion criteria

1‐ Studies that scored less than 5 points for study quality were excluded from the meta-analysis. 2‐ Duplicate publications, reviews, animal researches, case reports, in-vitro, and in-silico studies were removed from the meta-analysis.

### Study selection

First, the full text or summary of articles, documents, and reports were extracted. Then, duplicates and irrelevant articles were excluded from this study. Next, the case reports and review studies were removed from this meta-analysis. Finally, data were extracted from full‐text articles based on inclusion and exclusion criteria.

### Quality evaluation

In this study, the risk of bias assessment was carried out by two researchers (H.J. and T.M) independently, and any disagreements in this step were resolved by the supervisor (M.M). In order to evaluate the quality of articles the Newcastle–Ottawa scale (NOS) checklist was applied. Based on this checklist, nine questions assigning a score and covering the type of study, sample size, study objectives, study population, sampling method, data analysis method, presentation of findings in an appropriate way, and presentation of results based on objectives were designed. Only the studies scored at least 5 points were included the study [9, 10].

### Data extraction

Data extraction based on article title, first author’s name, year of study, place of study, type of study, total number of samples, average and standard deviation of zinc, iron, B12, folic acid, ferritin, and Hb indicators. Moreover, number of people with normal and abnormal zinc, iron, B12, folic acid, ferritin, and HB indicators in the case and control groups were entered into the excl.

### Data analysis

Data analysis was performed with Stata software Ver. 11. According to the hematological parameters data (quantitative or qualitative), two indices of standardized mean difference (SMD) and odds ratio were estimated. We examined SMD, number of samples, means and standard deviation of the hematological indices for the case and control groups separately. It should be noted that if a study reported the median, the first and third quartiles instead of the mean and standard deviation, the required index was calculated using the formula mentioned in Wan et al.’s study [11]. Using Methane’s formula, random effect model and Cohen’s coefficient, the standardized mean difference was estimated for each hematological parameter with a 95% confidence interval. The criteria for judging the significance of the SMD of hematological indices between two groups (case and control) is not including zero between the upper and lower confidence intervals of the standardized mean difference. In order to estimate the odds ratio using a two-by-two table, the number of people with and without hematological deficiencies in two groups was extracted from each of the primary studies. Using Methane, random effect model and inverse variance, the odds ratio was estimated for each hematological parameter with 95% CI [12].

The criteria for judging the significance of the odds ratio of hematological indicators between two groups is not including the number one between the upper and lower confidence interval of the standardized mean difference. Heterogeneity between the results of primary studies was evaluated with I-square index and publication bias was performed by Egger’s test [13]. Sensitivity analysis was also done to check the effect of each of the primary studies on the overall estimate.

## Results

**Fig. 1:**
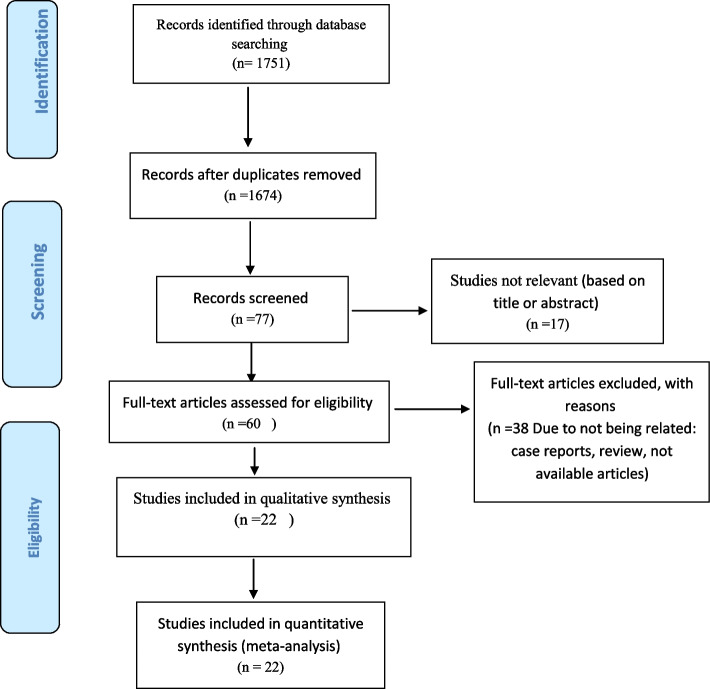
Process for searching and selecting primary studies

At first, according to the search strategy in the mentioned databases, 1751 articles were found. Among the primary articles 1674 duplicate manuscripts were removed using End Note software. Then, by screening the documents based on the title and abstract, 17 article were excluded based on the irrelevant title or abstracts. After that, 38 articles were omitted due to review, case report and unavailability. Finally, 22 articles was checked with the NOS checklist, and entered into this meta-analysis (Fig. 1).

The characteristics of the included articles are shown in Table 2. The year of published articles was varied from 2006 to 2023. These studies were conducted in Iraq, China, India, Iran, Jordan, Indonesia, Pakistan, Poland, UAE, Spain, Taiwan, and Turkey. The risk of bias for the results are presented in Table 3. This evaluation is done with the NOS checklist. Scores of risk of bias among studies that met the inclusion criteria varied from 6 to 9.

**Table 2:** Characteristics of primary studies

Author	Location	Type study	Case sample size	Control sample size
G S Ozler (2014) [14]	Turkey	case control	25	25
Nabiha Farasat Khan (2013) [15]	Pakistan	case control	60	60
Pia Lopez-Jornet(2014) [16]	Spain	case control	92	94
Kamran Sari (2016) [17]	Turkey	case control	195	217
Zuzanna Slebioda (2018) [18]	Poland	case control	71	70
Moin Sabeer Tidgundi(2017) [19]	India	case control	64	60
Zuzanna Ślebioda (2017) [20]	Poland	case control	75	72
Funda Tamer (2019) [21]	Turkey	case control	20	20
Vandana Sharma (2022) [22]	India	case control	50	50
Subhasish Mustafi (2021) [23]	India	case control	40	40
Song€ul Yılmaz (2020) [24]	Turkey	case control	108	57
Saime Sagiroglu (2018) [25]	Turkey	case control	175	175
Hatice Kaya Ozden (2021) [26]	Turkey	case control	81	69
Samar Z. Burgan(2006) [27]	Jordan	case control	143	143
Nurdiana, Pocut Astari (2018) [4]	Indonesia	case control	59	60
Zhe-Xuan Bao (2017) [28]	China	case control	517	187
Shaheen Ali Ahmed (2021) [29]	Erbil	case control	50	50
Suhail H. Al-Amad (2019) [30]	Sharjah United Arab Emirates	case control	52	52
Noor S. Mohammed Ali (2022) [31]	Baghdad	case control	30	30
Fateme Arbabi-Kalati (2014) [32]	Iran	case control	30	30
Neda Babaee (2015) [33]	Iran	case control	28	28
Andy Sun (2014) [34]	Taiwan	case control	273	273

**Table 3:** The results of the risk of bias assessment of each of the primary studies included in the study

First author (reference)	**Selection**				**Comparability**	**Exposure**			**Score**
	Is the case definition adequate?	Representativeness of the cases	Selection of Controls	Definition of Controls	Comparability of cases and controls on the basis of the design or analysis	Ascertainment of exposure	Same method of ascertainment for cases and controls	Non-Response rate	
G S Ozler (2014) [14]	-	*	*	*	-	*	*	*	6
Nabiha Farasat Khan (2013) [15]	*	*	*	*	**	*	*	*	9
Pia Lopez-Jornet(2014) [16]	*	*	*	*	**	*	*	*	9
Kamran Sari (2016) [17]	*	*	*	*	**	*	*	*	9
Zuzanna Slebioda (2018) [18]	*	*	*	*	**	*	*	*	9
Moin Sabeer Tidgundi(2017) [19]	*	*	*	*	**	*	*	*	9
Zuzanna Ślebioda (2017) [20]	*	*	*	*	-	*	*	*	8
Funda Tamer (2019) [21]	-	*	*	*	**	*	*	*	8
Vandana Sharma (2022) [22]	*	*	*	*	**	*	*	*	9
Subhasish Mustafi (2021) [23]	*	*	*	*	**	*	*	*	9
Song€ul Yılmaz (2020) [24]	*	*	*	*	**	*	*	*	9
Saime Sagiroglu (2018) [25]	*	*	*	*	**	*	*	*	9
Hatice Kaya Ozden (2021) [26]	*	*	*	*	**	*	*	*	9
Samar Z. Burgan(2006) [27]	*	*	*	*	**	*	*	*	9
Nurdiana, Pocut Astari (2018) [4]	*	*	*	*	**	*	*	*	9
Zhe-Xuan Bao (2017) [28]	*	*	*	*	**	*	*	*	9
Shaheen Ali Ahmed (2021) [29]	*	*	*	*	**	*	*	*	9
Suhail H. Al-Amad (2019) [30]	*	*	*	*	**	*	*	*	9
Noor S. Mohammed Ali (2022) [31]	-	*	*	*	**	*	*	*	8
Fateme Arbabi-Kalati (2014) [32]	-	*	*	*	**	*	*	*	8
Neda Babaee (2015) [33]	-	*	*	*	**	*	*	*	8
Andy Sun (2014) [34]	*	*	*	*	**	*	*	*	9

### Vitamin B12

**Fig. 2:**
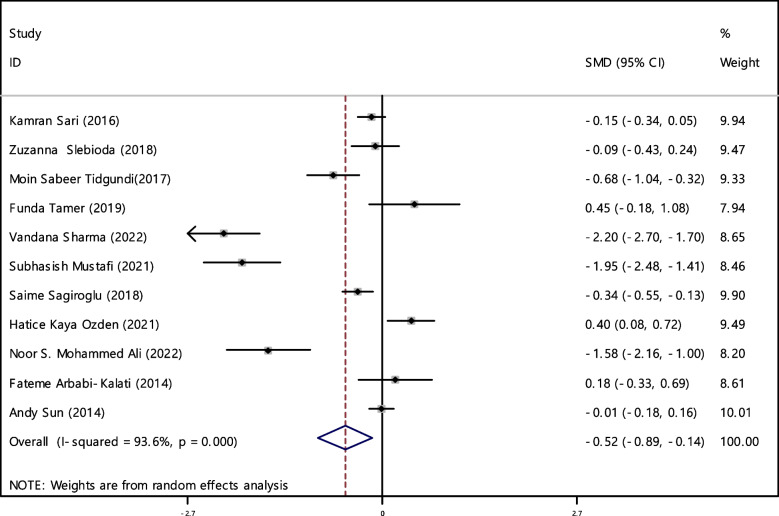
The standardized mean difference (SMD) of vitamin B12 between case and control groups according to primary studies and overall estimation

**Fig. 3:**
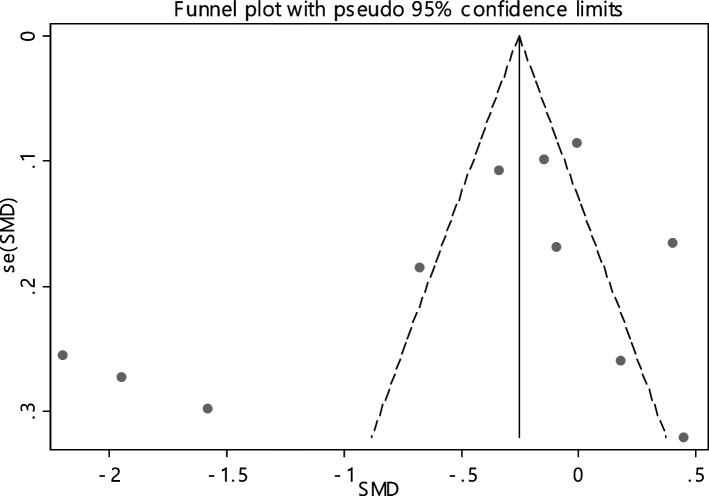
Funnel plot to investigate publication bias in estimating the standardized mean difference of vitamin B12 between case and control groups

**Fig. 4:**
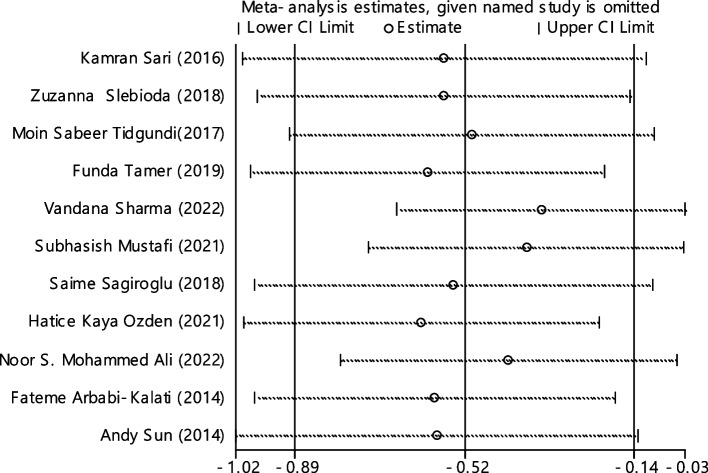
Sensitivity analysis in order to investigate the effect of each of the primary studies on the standardized mean difference of vitamin B12 between the case and control groups

In 11 documentations, the average of vitamin B12 level was compared between two groups. The total number of samples in the case and control groups was 1029 and 1024 respectively (Table 4). In 54.5% of documents (6 out of 11), the relationship between vitamin B12 and RAS was significant. By combining the results of 11 documents using the random effect model, the SMD of vitamin B12 between the two groups estimated to be -0.52 (95% CI; -0.89, -0.14) (Fig. 2), which is statistically significant. The I-square heterogeneity index was also equal to 93.6% and there was no publication bias based on Egger test (Fig. 3). The results of the sensitivity analysis have also showed that the effect of each of the studies on the overall assessment was not significant (Fig. 4).

**Table 4:** Pooled estimate of the standardized mean difference of hematological parameters between case and control groups

Index	Number of evidence	Sample size case	Sample size control	Pooled estimate result		Publication Bias total (Egger test)		Sensitivity analysis result (Yes, No)^a^	Heterogeneity (I−square), %
				SMD (95% CI SMD)	Significant association of between group (Yes, No)	β	*P*-value		
B12	11	1029	1024	-0.52(-0.89, -0.14)	Yes	-4.28	0.129	No	93.6
Ferritin	7	428	412	-0.20(-0.51, 0.11)	No	-1.21	0.600	No	75.6
Folic acid	6	629	617	-0.42(-0.95, 0.11)	No	-5.15	0.199	No	94.2
HB	4	663	685	-0.58(-0.90, -0.27)	Yes	1.58	0.754	No	85.1
Iron	5	465	414	0.01(-0.12, 0.15)	No	2.29	0.215	No	0
Zinc	4	150	147	-0.33(-0.81, 0.14)	No	-4.45	0.255	No	72.3

^a^Is there a significant difference in the impact of each of the primary studies on the overall estimate?

**Fig. 5:**
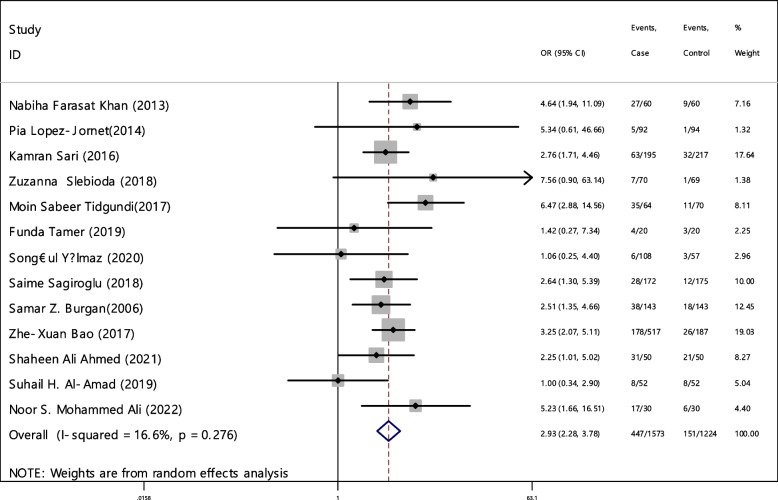
Odds ratio of vitamin B12 with 95% confidence interval according to primary studies and overall estimate

**Fig. 6:**
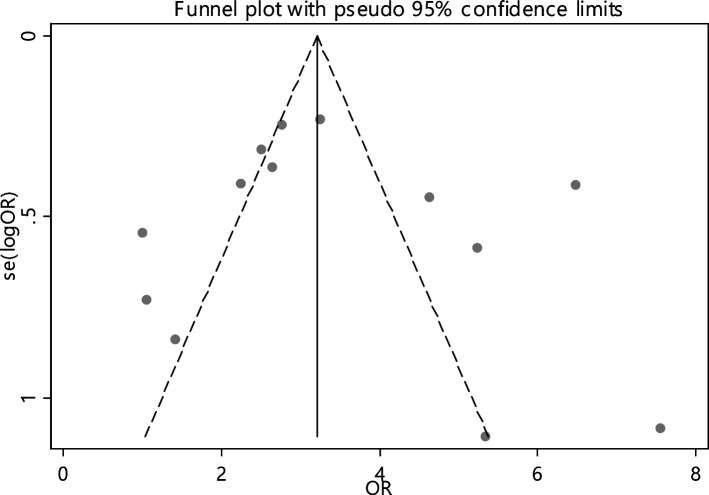
Funnel plot to investigate publication bias in vitamin B12 odds ratio estimates between case and control groups

**Fig. 7:**
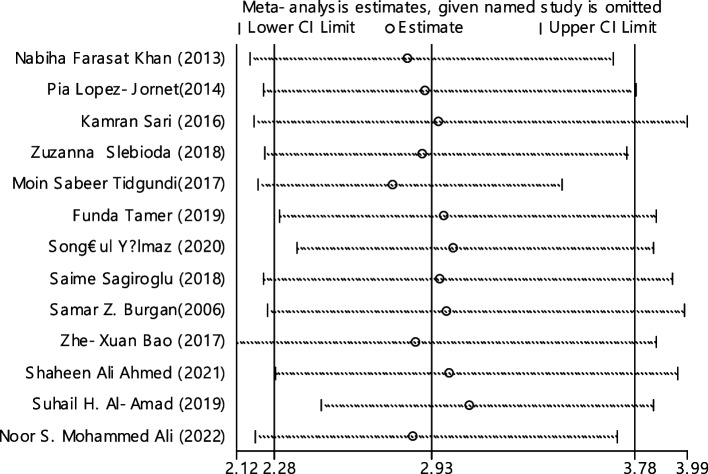
Sensitivity analysis in order to investigate the effect of each of the primary studies on the odds ratio of vitamin B12 between the case and control groups

In 13 articles the odds ratio was estimated in order to compare the vitamin B12 deficiency in two groups. The total samples in the case and control groups was 1573 and 1224 respectively (Table 5). In 61.5% of documents (8 out of 13), the relationship between vitamin B12 deficiency and RAS was significant. The combination of 13 results using the random effect model indicated that, patients with vitamin B12 deficiency are 2.93 times more suitable to develop RAS (95% CI; 2.28, 3.78), which is statistically significant. The I-square heterogeneity index was 16.6% (Fig. 5) and no publication bias was seen (Fig. 6). Also, the results of the sensitivity analysis showed that the impact of each of the studies on the overall assessment was not significant (Fig. 7).

**Table 5:** Pooled estimate of the odds ratio of hematological parameters between case and control groups

Index	Number of evidence	Sample size case	Sample size control	Pooled estimate result		Publication Bias total (Egger test)		Sensitivity analysis result (Yes, No)^a^	Heterogeneity (I−square), %
				OR (95% CI)	Significant impact of exposure on the index (Yes, No)	β	*P*-value		
B12	13	1573	1224	2.93(2.28, 3.78)	Yes	1.53	0.528	No	16.6
Ferritin	9	1144	819	2.50(1.48, 4.22)	Yes	3.15	0.690	No	63.2
Folic acid	8	1193	817	1.51(0.53, 4.29)	No	5.18	0.400	No	81.1
HB	9	831	855	2.14(1.38, 3.32)	Yes	3.32	0.396	No	53.3
Iron	5	459	413	1.46(0.70, 3.03)	No	4.57	0.136	No	66.8

^a^Is there a significant difference in the impact of each of the primary studies on the overall estimate?

### Ferritin

**Fig. 8:**
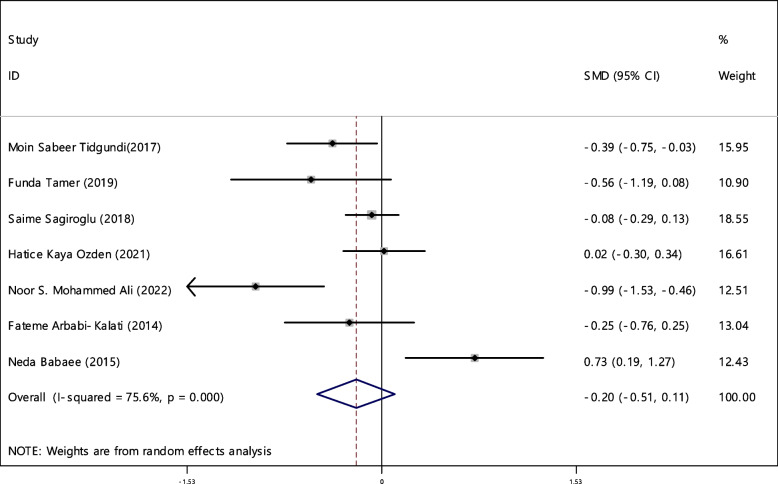
The standardized mean difference (SMD) of ferritin between case and control groups according to primary studies and overall estimation

**Fig. 9:**
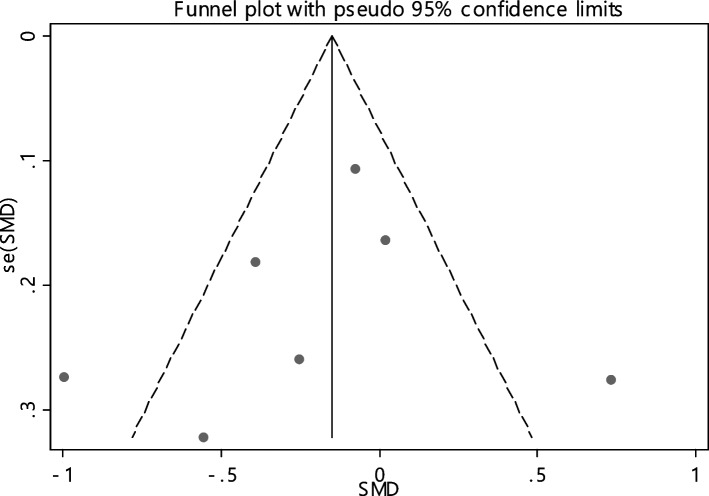
Funnel plot to investigate publication bias in estimating the standardized mean difference of vitamin ferritin between case and control groups

**Fig. 10:**
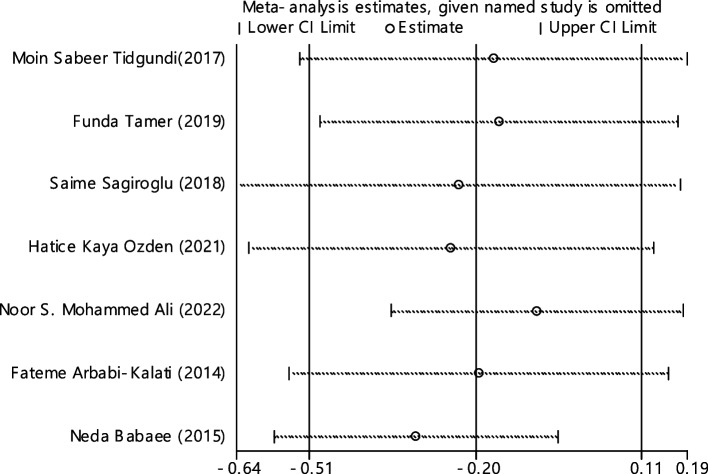
Sensitivity analysis in order to investigate the effect of each of the primary studies on the standardized mean difference of ferritin between the case and control groups

In 7 Articles, the average index of ferritin was compared between two groups. The sample size of case and control groups was 428 and 412 respectively (Table 4). In 3 out of 7 studies the relationship between ferritin deficiency and RAS was significant. By combining the results of 7 documents using the random effect model, the SMD of ferritin level between two groups estimated as -0.20 (95%; -0.51, 0.11), which was not statistically significant. The I-square heterogeneity index was 75.6% (Fig. 8) and according to the Egger test, publication bias was not observed (Fig. 9). Besides, the sensitivity analysis indicated that the impact of each of the studies on the overall assessment was not significant (Fig. 10).

**Fig. 11:**
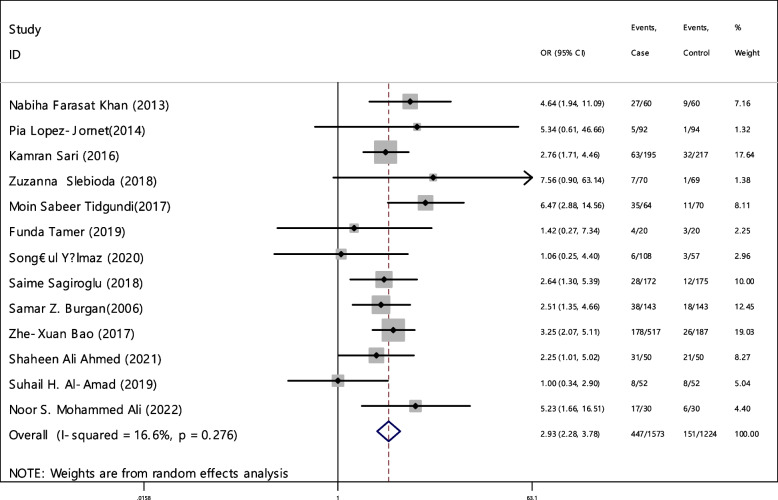
Odds ratio of ferritin with 95% confidence interval according to primary studies and overall estimate

**Fig. 12:**
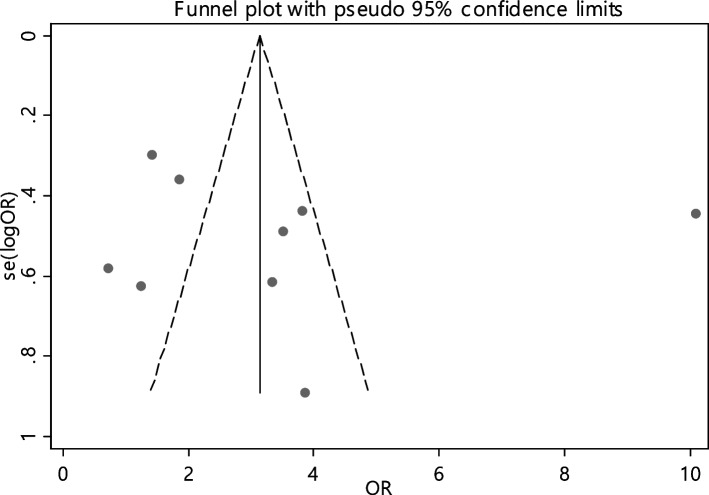
Funnel plot to investigate publication bias in ferritin odds ratio estimates between case and control groups

**Fig. 13:**
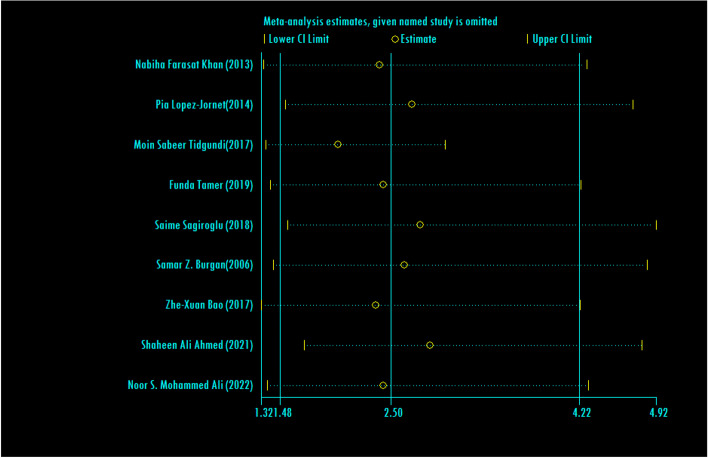
Sensitivity analysis in order to investigate the effect of each of the primary studies on the odds ratio of ferritin between the case and control groups

The odds ratio of ferritin deficiency was estimated in 1144 cases and 819 controls in 9 articles (Table 5). Among 9 investigated articles, 7 studies showed significant relationship between ferritin deficiency and developing RAS. The combined results of 9 documents indicated that odds ratio of ferritin deficiency 2.50 (95% CI; 1.48, 4.22) in case group compared to the control group, was statistically significant. The I-square heterogeneity index was 63.2% (Fig. 11) and according to the Egger test, there was no publication bias (Fig. 12). The results of the sensitivity analysis have also showed that the effect of each of the studies on the overall assessment was not significant (Fig. 13).

### Folic acid

**Fig. 14:**
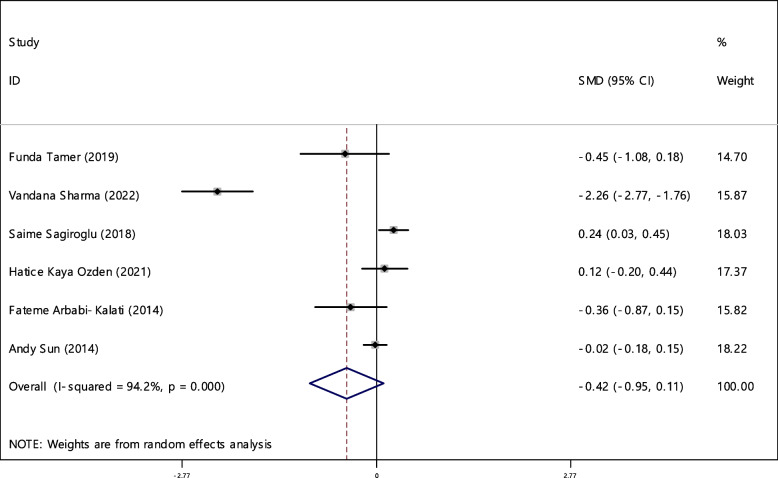
The standardized mean difference (SMD) of folic acid between case and control groups according to primary studies and overall estimation

**Fig. 15:**
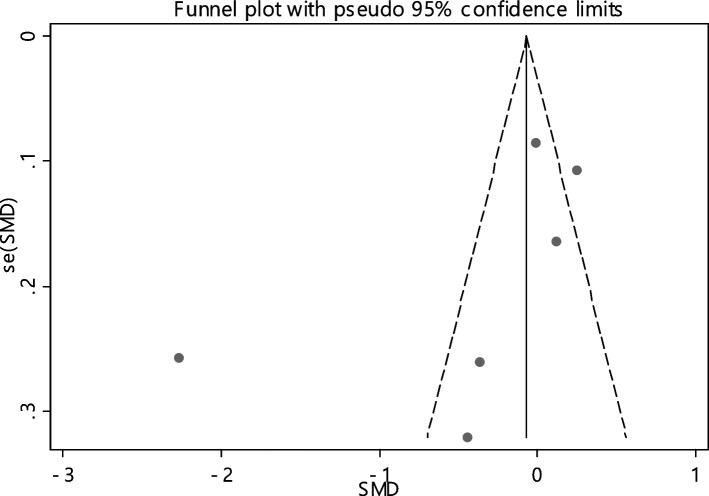
Funnel plot to investigate publication bias in estimating the standardized mean difference of folic acid between case and control groups

**Fig. 16:**
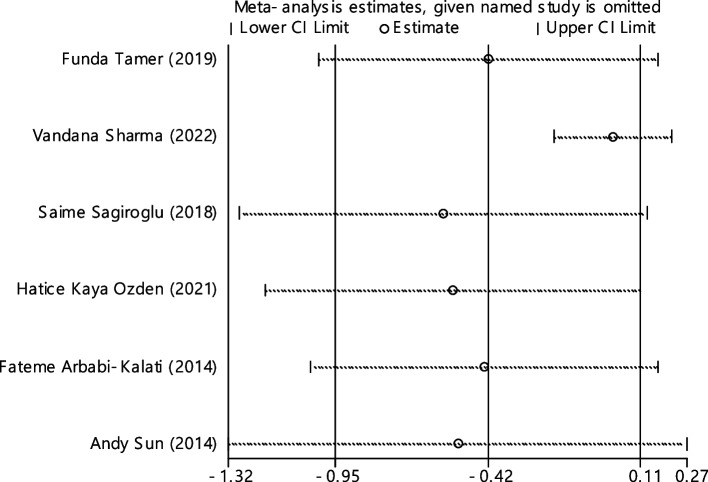
Sensitivity analysis in order to investigate the effect of each of the primary studies on the standardized mean difference of folic acid between the case and control groups

The average index of folic acid was compared between two groups including 629 cases and 617 controls in 6 documents (Table 4). In 33.3% of the documents (2 out of 6), the relationship between folic acid deficiency and RAS was significant. The SMD of folic acid between all samples of two groups estimated as -0.42 (95% CI, -0.95, 0.11) which was not statistically significant. The I-square heterogeneity index was equal to 94.2% (Fig. 14), and there was no publication bias (Fig. 15). The results of the sensitivity analysis have also showed that the effect of each of the studies on the overall assessment was not significant (Fig. 16).

**Fig. 17:**
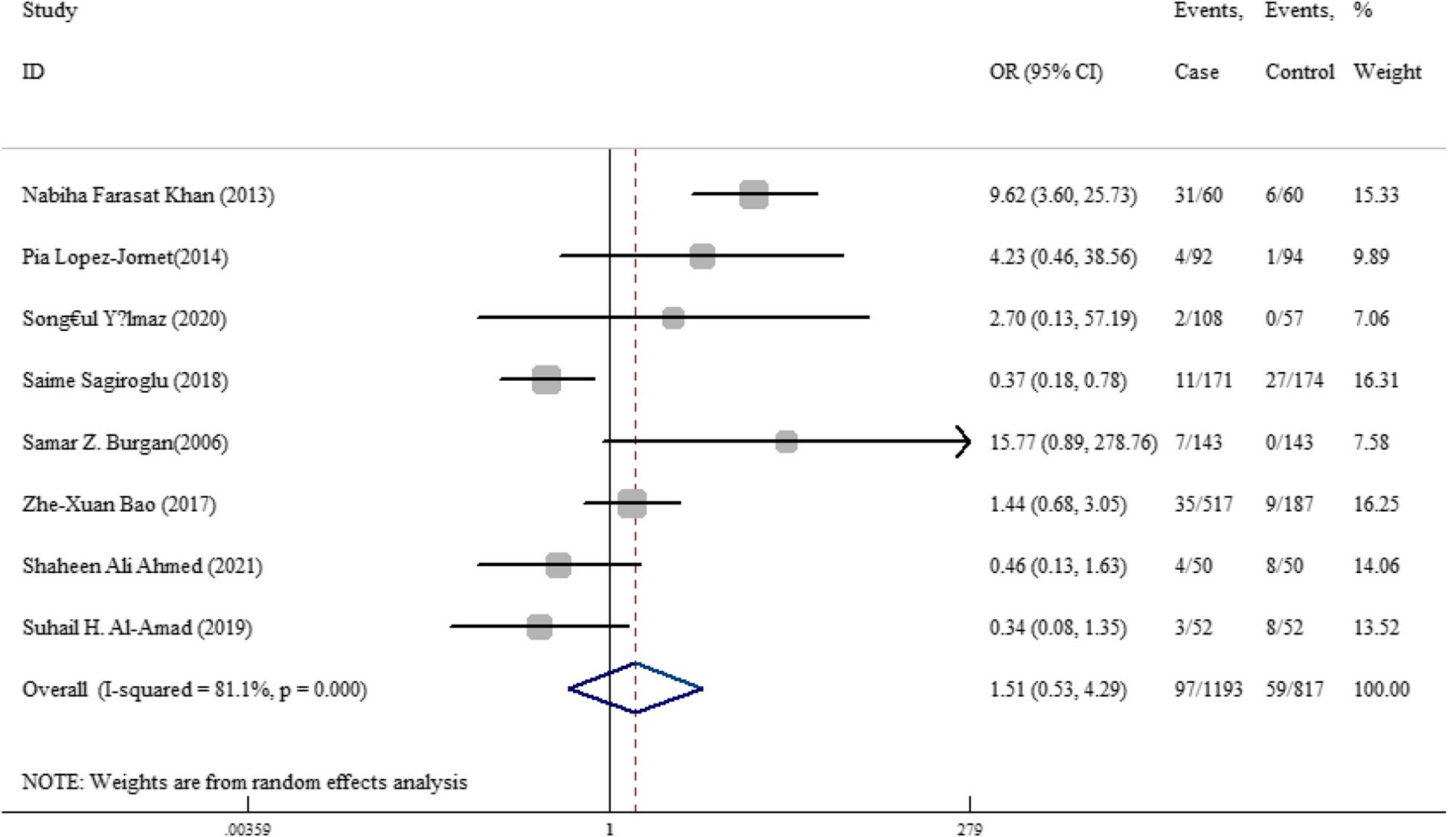
Odds ratio of folic acid with 95% confidence interval according to primary studies and overall estimate

**Fig. 18:**
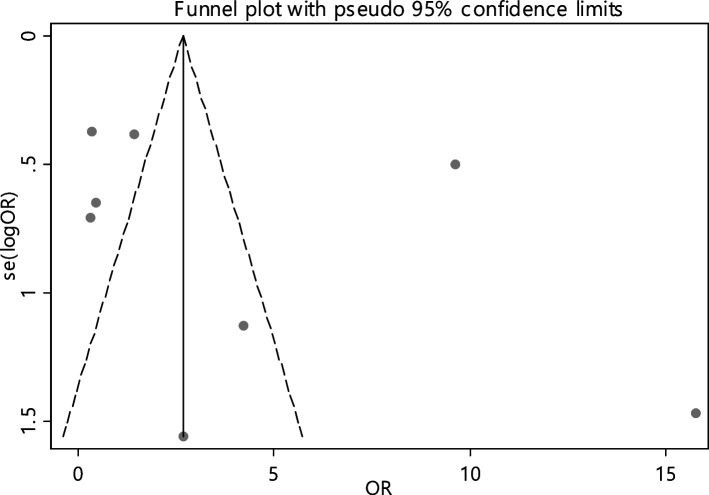
Funnel plot to investigate publication bias in folic acid odds ratio estimates between case and control groups

**Fig. 19:**
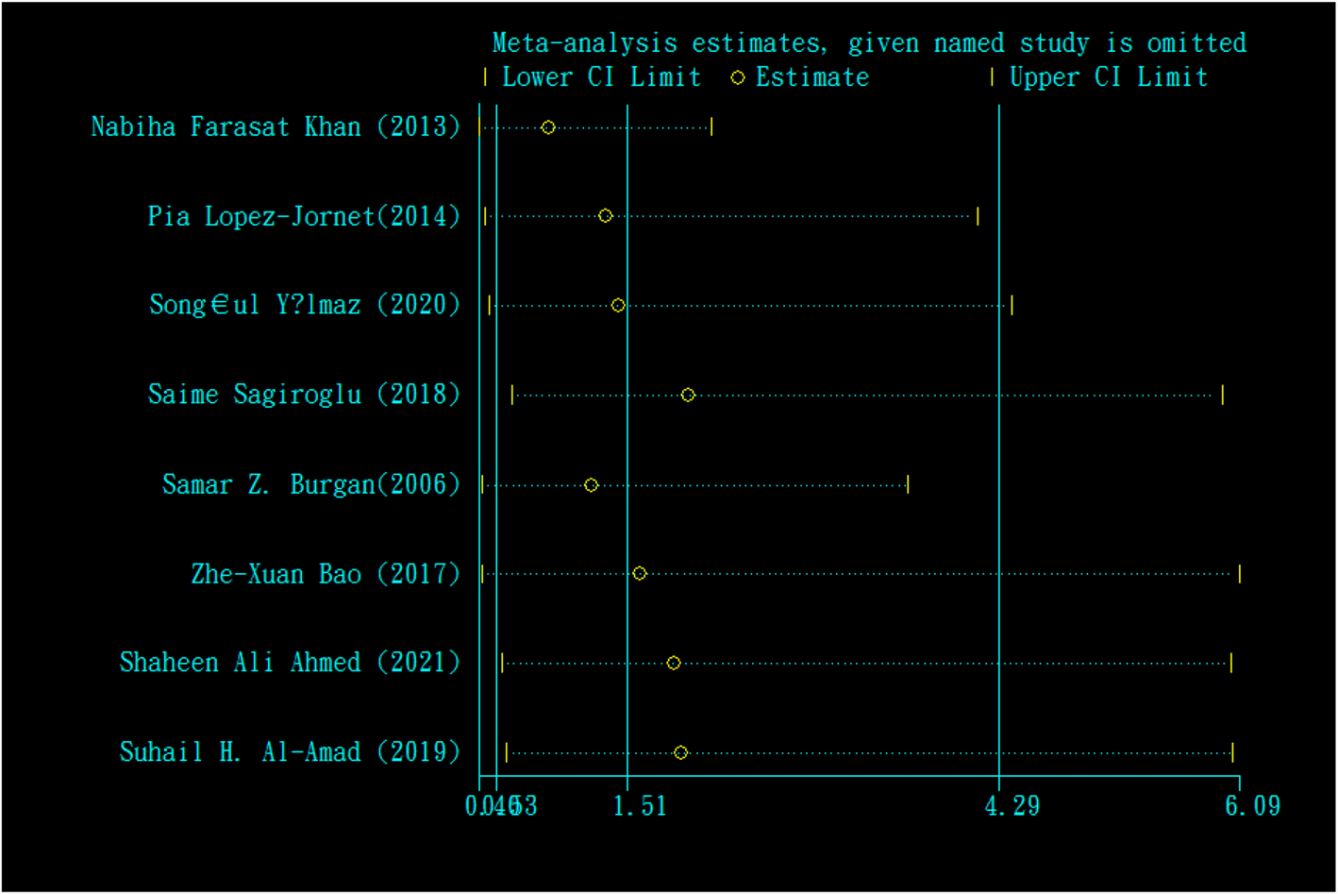
Sensitivity analysis in order to investigate the effect of each of the primary studies on the odds ratio of folic acid between the case and control groups

The odds ratio of folic acid deficiency in 1193 cases versus 817 controls was estimated in 8 documents (Table 5). Only in 25% of documents (2 out of 8), the relationship between folic acid deficiency and RAS was significant. The odds ratio of folic acid deficiency in the case group compared to the control group was 1.51 (95% CI; 0.53, 4.29), which was not statistically significant. The I-square heterogeneity index was 81.1% (Fig. 17), and there was no publication bias (Fig. 18); also, the impact of each of the studies on the overall assessment was not significant (Fig. 19).

### Hemoglobin (Hb)

**Fig. 20:**
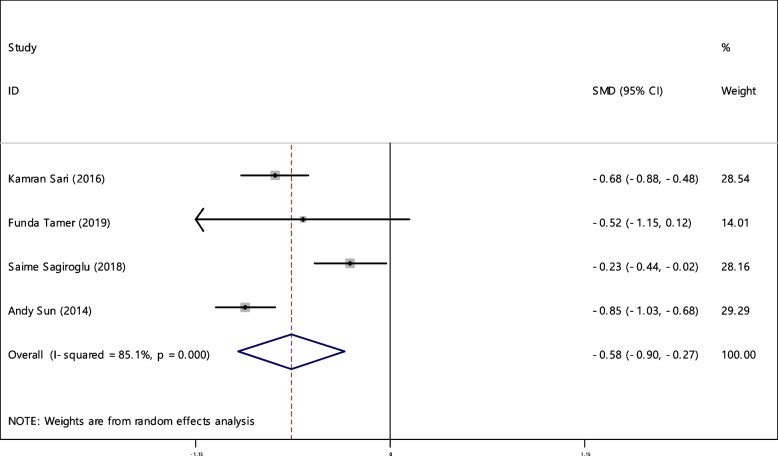
The standardized mean difference (SMD) of HB between case and control groups according to primary studies and overall estimation

**Fig. 21:**
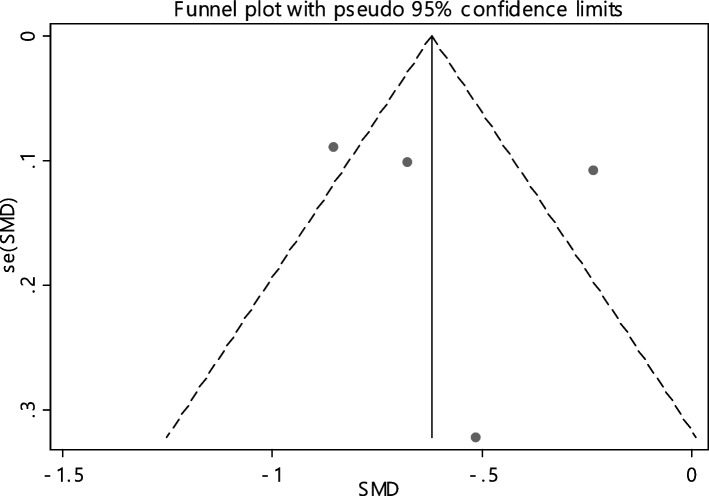
Funnel plot to investigate publication bias in estimating the standardized mean difference of HB between case and control groups

**Fig. 22:**
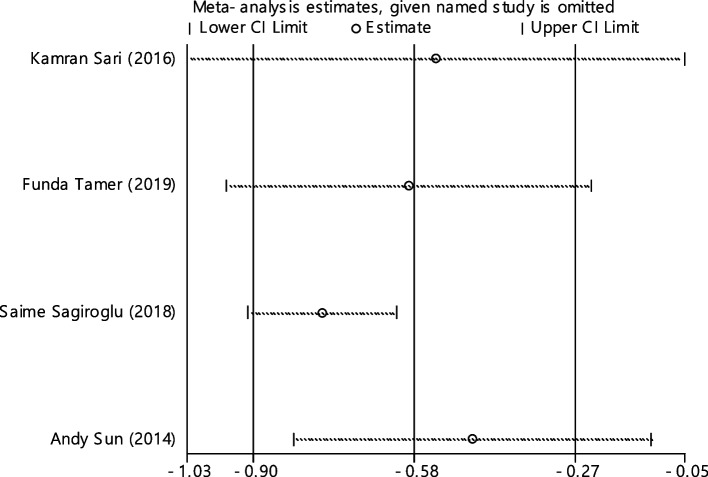
Sensitivity analysis in order to investigate the effect of each of the primary studies on the standardized mean difference of HB between the case and control groups

The average index of ferritin was compared between two groups including 663 cases and 685 controls in 4 articles (Table 4). In 75% of documents (3 out of 4), the relationship between reduced Hb level and RAS was significant. A statistically significant SMD of Hb level between the two groups was observed -0.58 (-0.90, -0.27). The I-square heterogeneity index was equal to 85.1% (Fig. 20) and it was no publication bias (Fig. 21). Also, the impact of each of the studies on the overall assessment was not significant (Fig. 22).

**Fig. 23:**
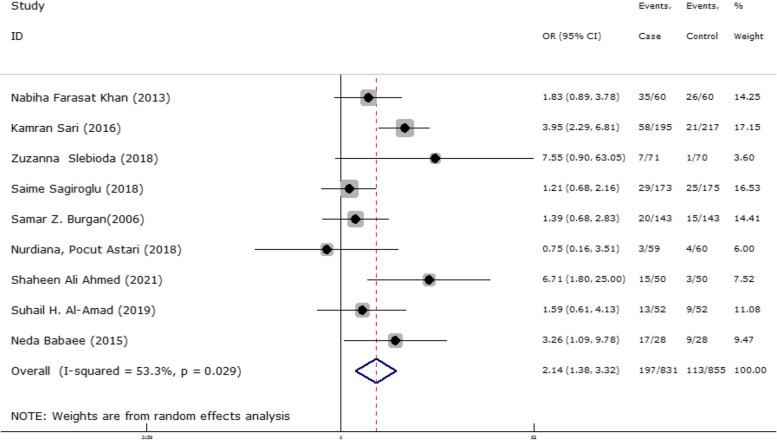
Odds ratio of HB with 95% confidence interval according to primary studies and overall estimate

**Fig. 24:**
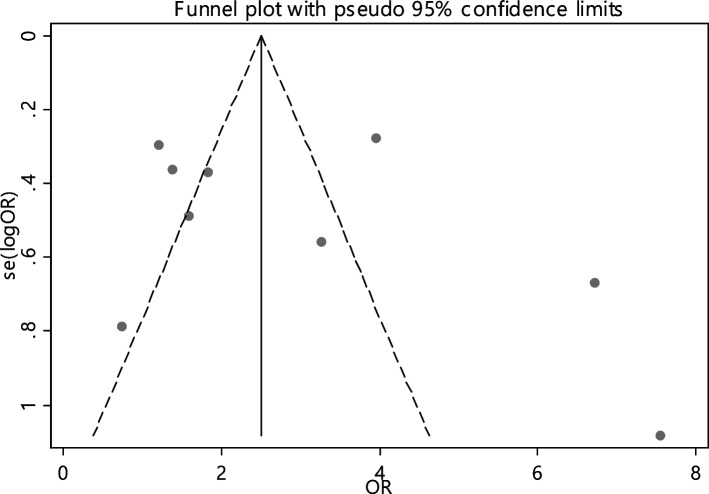
Funnel plot to investigate publication bias in HB odds ratio estimates between case and control groups

**Fig. 25:**
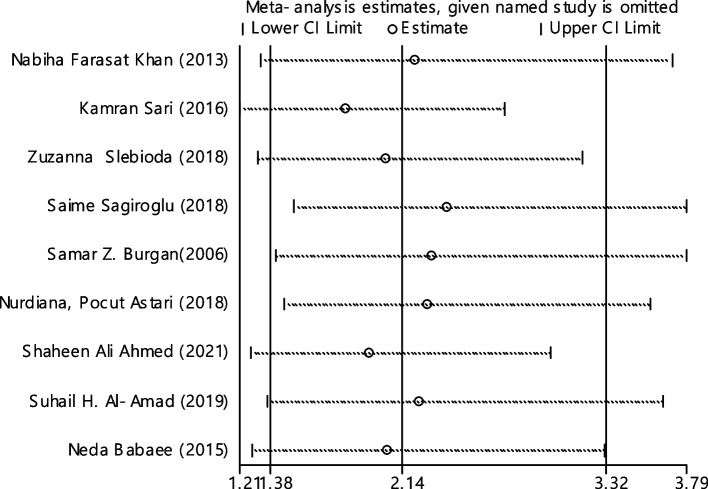
Sensitivity analysis in order to investigate the effect of each of the primary studies on the odds ratio of HB between the case and control groups

In 9 articles odds ratio of Hb level in developing RAS was estimated in 831 cases versus 855 controls (Table 5). In 3 out of 9 documents (33.3%), the relationship between Hb level and RAS was significant. The overall results of these 9 articles indicated that the odds ratio of reduced Hb level in the case group compared to the control group estimated 2.14 (95% CI; 1.38, 3.32), which was statistically significant. The I-square heterogeneity index was 53.3% (Fig. 23) and according to the Egger test, there was no publication bias (Fig. 24). Also, the results of the sensitivity analysis showed that the impact of each of the overall assessment studies was not significant (Fig. 25).

### Iron

In 5 studies the average index of iron was compared between two groups (465 cases and 414 controls) (Table 4). It was not found any association between iron deficiency and RAS.

**Fig. 26:**
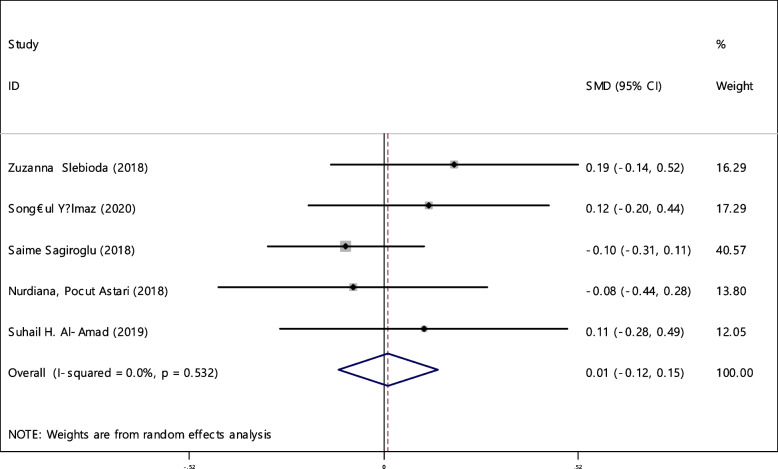
The standardized mean difference (SMD) of iron between case and control groups according to primary studies and overall estimation

**Fig. 27:**
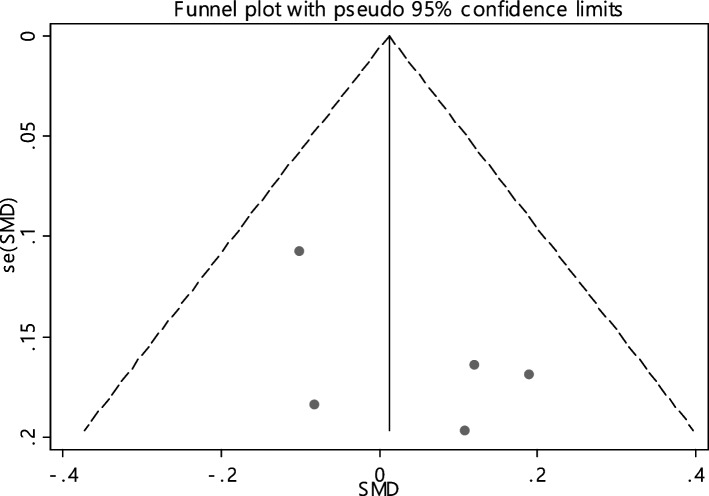
Funnel plot to investigate publication bias in estimating the standardized mean difference of iron between case and control groups

**Fig. 28:**
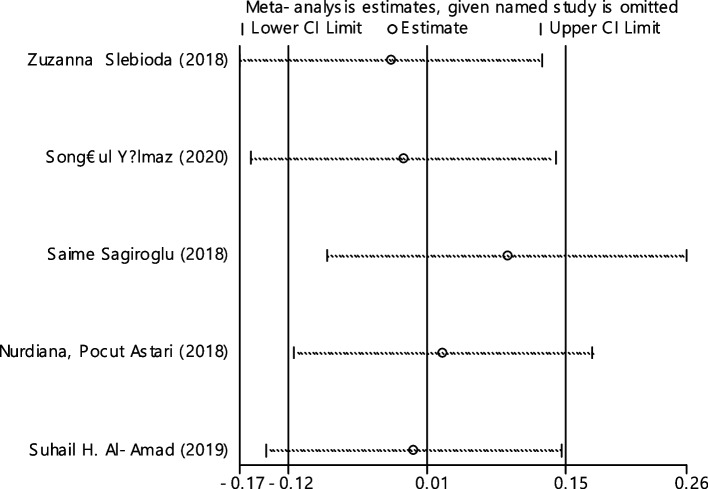
Sensitivity analysis in order to investigate the effect of each of the primary studies on the standardized mean difference of iron between the case and control groups

The combination of 5 documents indicated that the SMD of iron between the two groups was not statistically significant 0.01 (95% CI; -0.12, 0.15). The I-square heterogeneity index was zero (Fig. 26) and there was no publication bias (Fig. 27). Also, the effect of each of the studies on the overall assessment was not significant (Fig. 28).

**Fig. 29:**
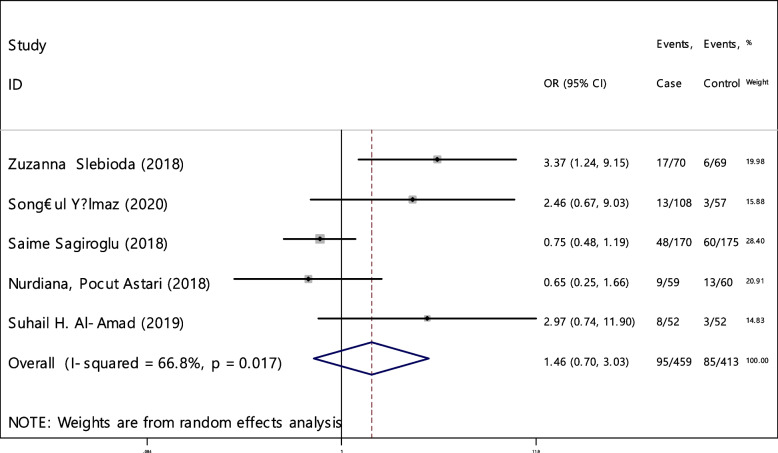
Odds ratio of iron with 95% confidence interval according to primary studies and overall estimate

**Fig. 30:**
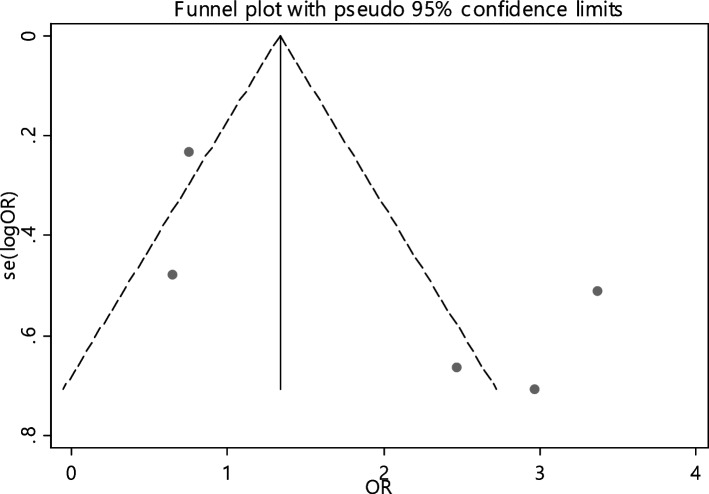
Funnel plot to investigate publication bias in iron odds ratio estimates between case and control groups

**Fig. 31:**
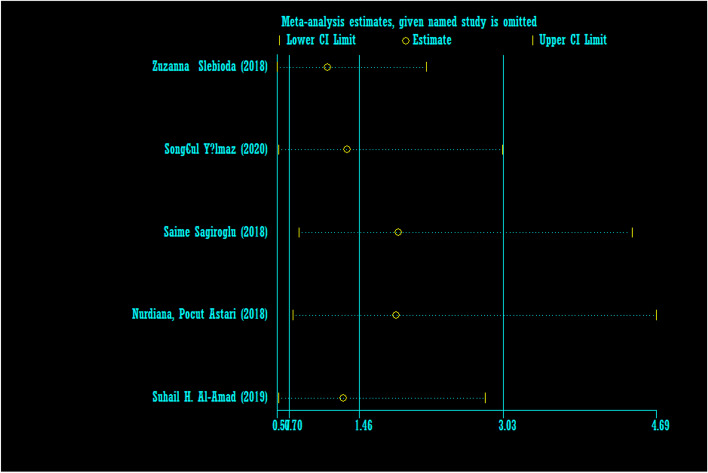
Sensitivity analysis in order to investigate the effect of each of the primary studies on the odds ratio of iron between the case and control groups

The odds ratio of iron deficiency in developing RAS between cases (459 patients) and controls (413 healthy) was calculated in 9 articles (Table 5). In only 20% of the documents (1 out of 5), the relationship between iron deficiency and RAS was significant. The random effect model analysis of these 5 articles showed that the odds ratio of iron deficiency in the case groupcompared to the control group with the 95% confidence interval was 1.46 (0.70, 3.03), which is statistically not significant. The I-square heterogeneity index of the articles was 66.8% (Fig. 29) and the Egger test showed no publication bias (Fig. 30). Also, the results of the sensitivity analysis showed that the impact of each of the overall assessment studies is not significant (Fig. 31).

### Zinc

**Fig. 32:**
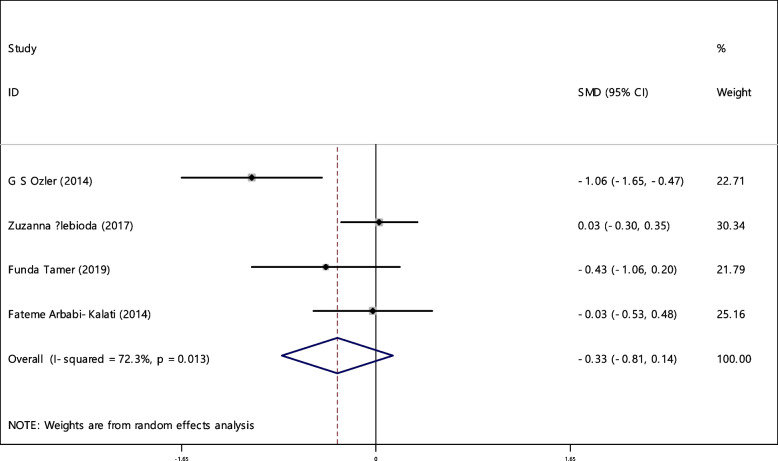
The standardized mean difference (SMD) of zinc between case and control groups according to primary studies and overall estimation

**Fig. 33:**
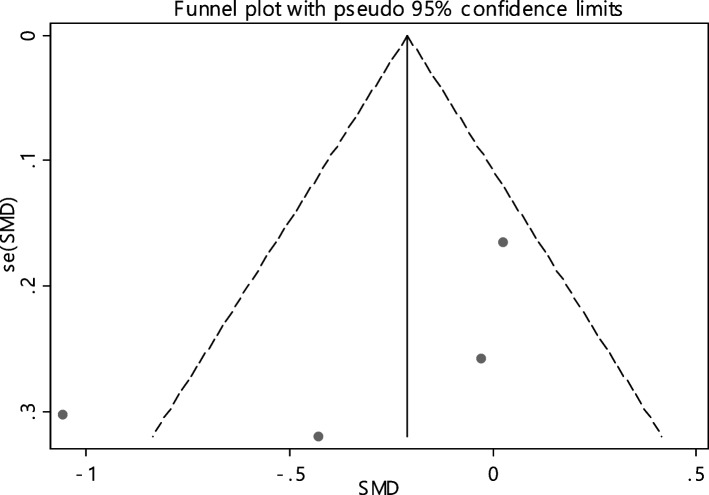
Funnel plot to investigate publication bias in estimating the standardized mean difference of zinc between case and control groups

**Fig. 34:**
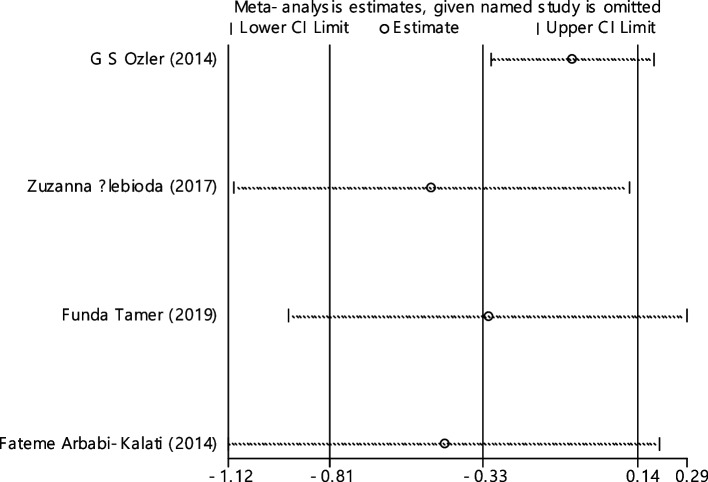
Sensitivity analysis in order to investigate the effect of each of the primary studies on the standardized mean difference of zinc between the case and control groups

The average index of zinc was compared between two groups (150 RAS patients and 147 controls). Based on the results of articles in 25% of the documents (1 out of 4), the relationship between zinc deficiency and RAS was significant. The random effect model results showed that the SMD of zinc with the 95% confidence interval between the two groups is estimated as -0.33 (-0.81, 0.14), which is not statistically significant. The I-square heterogeneity index was 72.3% (Fig. 32) and no publication bias was observed (Fig. 33). The results of the sensitivity analysis showed that the effect of each of the studies on the overall assessment was not significant (Fig. 34). It should be noted that the number of evidences to estimate the odds ratio in order to compare the zinc deficiency in both groups (case and control) was not sufficient.

## Discussion

The results of the meta-analysis indicated that the SMD of vitamin B12 serum and Hb levels in the case group was 52% and it was 58% lower than the control group. Also, our result showed that the odds ratio of B12, ferritin, and Hb levels below normal in the case group was 2.93, 2.50, and 2.14 times more than the control group.

Because of recurrent and painful intraoral mucosal lesions and discomfort eating, drinking, and speaking, RAS influence on the quality of life of affected patients. Some medications are prescribed for most of the patients with RAS in order to relieve the pain without doing investigations [29]. However, the exact role of vitamin B12 deficiency in creating RAS is still unknown but some research reported that iron, folate, and vitamin B12 deficiencies plays a significant role in developing RAS. According to the study of Wray et al. [35] hematinic deficiencies was reported up to 21% of adult patients with RAS, and replacement therapy was effective among 59% of the patients and 28% showed significant improvement. However, other studies have indicated that although serum iron, folate, or vitamin B12 levels were different between RAS and control group, the replacement of the deficient factors had not always been lead to effective treatment [35–38]. Moreover, patients with vitamin B12 or folate deficiency showed rapid improvement to replacement therapy in comparison to RAS patients with iron deficiency that had shown a less remarkable response [37–39]. The results of the present study indicated that serum level of vitamin B12 deficiency was significantly different between RAS and healthy individuals. This highlights the important role of vitamin B12 deficiency on occurring RAS.

In the present study, Hb level was another factor that had a significantly lower frequency in RAS patients than controls. The same results was also reported in the study of Rodriguez‑Archilla et al. that showed RAS patients had 17.30 times lower than Hb levels (< 14 g/dL) compared with healthy individuals [40]. They reported that lower Hb concentrations in 0.85 g/dL on average were found in patients with RAS that is significant statistically different in comparison to the controls. It is argued that the lack of Hb leads to reduction in the capacity of the blood to transport oxygen to the oral tissues, such as epithelial atrophy that may induce developing RAS lesions [34]. A meta-analysis conducted by Rodriguez‑Archilla et al. at 2019 [40] reported that the Hb (OR: 17.30), iron (OR: 6.67), folic acid (OR: 4.98), vitamin B12 (OR: 3.99), and ferritin (OR: 2.86) deficiencies increase the risk of developing RAS which was compatible with the results of present study (Hb (OR: 2.14), iron (OR: 1.46), folic acid (OR: 1.51),(vitamin B12, OR: 2.93), and ferritin (OR: 2.5)).

## Data Availability

All data generated or analyzed during this study are included in this published article [and its supplementary information files].
